# Spatial Distribution of Lead in Sacramento, California, USA

**DOI:** 10.3390/ijerph120303174

**Published:** 2015-03-17

**Authors:** Michael J. Solt, Daniel M. Deocampo, Michelle Norris

**Affiliations:** 1 Department of Geology, California State University Sacramento, 6000 J Street, Sacramento, CA 95819, USA; E-Mail: mjsolt100@gmail.com; 2 Department of Geosciences, Georgia State University, 24 Peachtree Center Avenue, Kell Hall 340, Atlanta, GA 30303, USA; 3 Department of Mathematics and Statistics, California State University Sacramento, 6000 J Street, Sacramento, CA 95819, USA; E-Mail: Norris@csus.edu

**Keywords:** lead, Pb, urban

## Abstract

Chronic exposure to lead remains a health concern in many urban areas; Sacramento, California is one example, with state surveillance data showing nearly 3% of screened children reported with blood lead levels over 4.5 μg/dL in 2009. To investigate the environmental exposure, 91 soil samples were collected and analyzed by ICP-AES and ICP-MS for 14 elements. An additional 28 samples were collected from areas of focus and analyzed by hand-held X-ray fluorescence spectrometry for Pb and Zn. Analysis of the metals data revealed non-normal distributions and positive skewness, consistent with anthropogenic input. In addition, high correlation coefficients (≥0.75) of metal concentrations in Cd-Pb, Cd-Zn, Pb-Zn, and Sb-Sn pairs suggest similarities in the input mechanisms. Semivariograms generated from Pb and associated metals reveal these metals to exhibit spatial correlation. A prediction map of lead concentrations in soil was generated by ordinary kriging, showing elevated concentrations in soil located in the central, older area of Sacramento where historic traffic density and industrial activity have been historically concentrated. XRF analysis of Pb and Zn from additional samples verifies elevated concentrations in the central areas of Sacramento as predicted.

## 1. Introduction

Anthropogenic contamination of soils from lead and other metals is a common problem in urban areas worldwide. Lead-contaminated urban soils are known to cause elevated blood-lead levels in children due to exposure both inside and outside of residences [1,2,3,4,5]. Lead-contaminated house dust, a major source of lead exposure for children [1,3], often originates from re-suspension of dust as well as outside dust tracked in from lead-contaminated soil [2]. Recent studies reveal significant cognitive impairment can occur at very low blood-lead levels, even below the current post-abatement clearance standards of 5 μg/dL [2,6,7]. Urban areas around the world are therefore confronted with the legacy of lead contamination that in many cases represents a chronic source of lead exposure for infants and toddlers, who are the most vulnerable [8]. The spatial link between diffuse soil Pb content and the blood lead levels of resident children is well known from several major cities in the U.S. and elsewhere [9], and recent work has shown the importance of the seasonal resuspension of soil Pb in controlling exposure and blood lead levels among children [10].

Sacramento is the capital of California, with a population of about 450,000, an aging housing stock containing lead paint, a legacy of transportation-related lead emissions [8], and a chronic caseload of lead blood poisoning in children [11,12,13]. Sacramento County had 1,418,788 residents in 2010 [14]. A 2005 review of Sacramento County health records reported 257 cases of lead poisoning from 1989–2004 [15,16], and the State of California [17] found in 2007, 2008, and 2009 that approximately 6.0%, 1.7%, and 2.4%, respectively, of children screened in Sacramento County had blood lead levels greater than 4.5 μg/dL.

Natural soils of Sacramento are generally classified as alfisols and entisols developed on quartzo-feldspathic river sediment and basement rock of the Sierra Nevada foothils, with moderate base cation content and abundant aluminum-rich clay minerals and Fe-oxyhydroxides [18]. Although no regional investigation has been carried out in the area specifically addressing lead contamination, previous studies have sampled soils and sediment of the area for heavy metal concentrations. These works include U.S. Geological Survey (USGS) studies of metal loading in the Sacramento River [19] and the U.S. Department of Energy National Uranium Resource Evaluation Hydrogeochemical Stream Sediment Reconnaissance (NURE-HSSR) studies revisited and published by the USGS [20]. These and other studies show that natural lead levels in Sacramento area soils are low, generally <20 mg/kg [21]; elevated levels are therefore due to some combination of anthropogenic inputs such as transportation, industrial, and paint sources. With recent changes in California lowering the soil guideline limit to 80 mg/kg Pb, it is important to newly assess the degree to which ambient urban environments exceed this limit [22].

The purpose of this study is to test for systematic spatial distribution and possible relationships to anthropogenic sources of lead and statistically associated heavy metals. We use kriging to aid in the visualization of the spatial distribution of lead concentrations and predict concentrations at unsampled locations, following the approach of other investigators, e.g., [23,24,25].

## 2. Methods

### 2.1. Sample Collection and Analysis Methods

**Figure 1:**
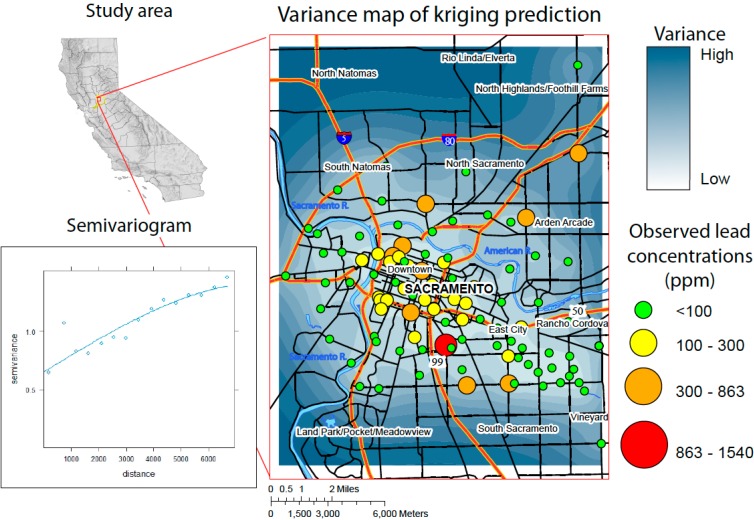
Map of the study area, with observed soil Pb concentrations, mapped variance, and the associated semivarigram. The semivariogram indicates that differences in Pb concentration are low at small distances, and they increase with distance, confirming that the kriging method is appropriately applied. The variance map of kriging predictions displays filled contours exhibiting the difference between the predicted (kriged) surface and the observed concentration values. Variance is lowest in the central, downtown areas of Sacramento and increase outward where the sample sites are more isolated. It is apparent that observed values at sample sites with nearby neighbors are more likely to match the predicted values.

Ninety-one soil samples were collected in the vicinity of Sacramento and analyzed for multiple trace metals, with an additional 10 samples analyzed only for cadmium, lead, and zinc (see Figure 1 for site locations). These samples were selected based on public accessibility to land; their location in a passive geomorphologic setting; and sites with little vegetation. Their distribution throughout Sacramento was chosen by their proximity to major roads, industrial areas, and their proximity to each other and previously collected samples. A Garmin Etrex GPS unit catalogued the latitude and longitude coordinates with respect to the 1983 North American Datum. Samples were collected in a 0.1 m^2^ area to a depth of 5 cm. The soil samples were collected with a stainless steel hand-spade and placed into a plastic bag. Aliquots of soil collected from the plastic bags were heated on Pyrex dishes at 80 °C overnight with the exception of the 30 samples collected for analysis by XRF, which were not heated. The dried samples were sieved to remove pebbles and debris. Approximately 250 g of dried and sieved subsample were shipped to ALS Chemex (Sparks, NV, USA) for pulverization and analysis by 4-acid digestion (perchloric, nitric, hydrofluoric, and hydrochloric) and a combination of ICP-AES and ICP-MS analyses (Table 1; ME-MS61 method). ME-MS61 is the method name for ALS Chemex ultra-trace level analytical procedure using a combination of four-acid digestion, ICP-AES, and ICP-MS analyses, referred to below as “MEMS”.

In addition to the samples analyzed by ICP methods, an additional 30 samples were selected for hand-held X-ray fluorescence spectroscopy analysis to verify the validity of the ICP-based predictions produced by kriging. The hand-held XRF was an Innov-X α-4000 X-Ray Fluorescence spectrometer at Georgia State University using tungsten radiation and an energy-dispersive spectrometer. Samples for XRF analysis were sieved and analyzed in triplicate; concentrations were reported with a standard deviation (Table 2). XRF standardization was tested using NIST standard reference materials 2787 [26]. The median concentration is used to calculate results of XRF samples.

### 2.2. Statistical Methods

The data from the ME-MS61 analysis were tested for spatial correlation and kriged using the statistical computing software R [27]. To optimize the prediction maps output by the kriging calculations, a spherical model was fit to the estimated semivariogram. The lag size and maximum range varied among metals used in semivariogram calculations. Next, for metals exhibiting spatial correlation, the ordinary kriging calculations were applied to a predefined grid using the log of the metal concentrations and the semivariogram parameters discussed earlier. Details of the kriging methods are discussed in [12]. Factor analysis was also performed using the same software, generating a Maximum Likelihood Estimation [12,27].

## 3. Results

### 3.1. Analytical Results

#### 3.1.1. Elemental Analysis

**Figure 2:**
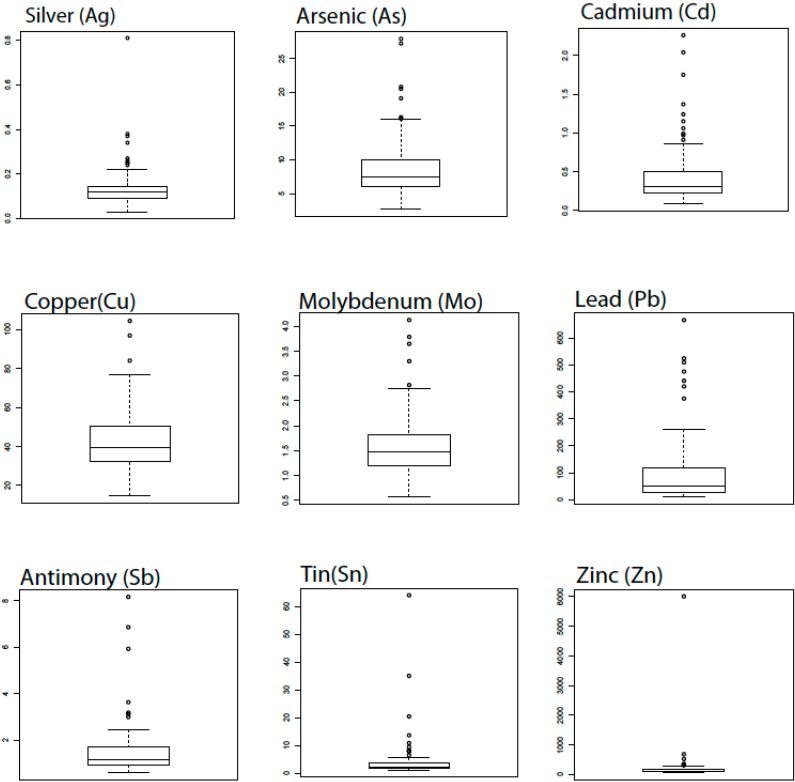
Boxplots of metal concentrations in mg/kg (or parts per million, ppm), showing non-normal distributions. Non-normal distributions with positive skewness exhibit elevated concentrations occur across all background levels. This is an indication that the elevated concentrations are of anthropogenic origin. The center line represents the average data value; top and bottom box edges represent the 25th and 75th percentiles, respectively; the top and bottom whiskers mark the 10th and 90th percentiles, respectively.

The analytical results for Pb are variable for both ICP-AES and hand-held XRF. Lead concentrations measured by ICP-AES range from 10.6 to 1540 mg/kg, averaging 128 mg/kg (σ = 204.9). Lead concentrations measured by hand-held XRF range from 0 to 750 mg/kg, averaging 171 mg/kg (σ = 141). A summary for the results for the elemental analysis of Ag, As, Bi, Cd, Cu, Mo, P, Pb, S, Sb, Sc, Sn, W, and Zn by the ME-MS61 are reported in milligrams per kilogram (mg/kg) except sulfur, which is reported by weight percent in Table 1. Box plots of Ag, As, Cd, Cu, Mo, Pb, Sb, Sn, and Zn (Figure 2) show non-normal distributions. A summary for the results for the elemental analysis of lead and zinc by hand-held XRF are reported in Table 2. Ten duplicate samples were analyzed by ME-MS61 and XRF methods for lead and zinc. Comparison of lead and zinc concentrations for 10 samples by both ICP-AES and XRF methods agreed with respective R-squared values of 0.95 and 0.93, with slightly higher lead concentrations detected by XRF.

Relative Percentage Differences (RPDs) were calculated for each variable based on eight duplicate analyses, following EPA analytical QA/QC protocols [28]. The least and the greatest values for each element were discarded, and the mean was taken of the remaining six RPD calculations, shown in Table 1. All values are well within acceptable RPD range of 20%, with the exception of Mo. The Mo data therefore should be treated with some care.

**Table 1:** Summary of elemental concentrations of soil samples collected in Sacramento, CA and analyzed by MEMS method (combined ICP-AES and ICP-MS). RPD = Relative Percent Differences based on 6 duplicate analyses for each analyte.

Analyte	Min.	Max.	Median	Mean	Standard Deviation	25th percentile	75th percentile	RPD
Ag (mg/kg) ^2^	0.03	0.81	0.11	0.14	0.09	0.09	0.14	18.7%
As (mg/kg) ^1^	2.7	27.9	7.5	8.9	4.7	6.0	10.0	5.7%
Bi (mg/kg) ^2^	0.07	0.67	0.15	0.16	0.09	0.12	0.18	21.3%
Cd (mg/kg) ^1^	0.08	2.63	0.34	0.49	0.47	0.23	0.56	11.4%
Cu (mg/kg) ^1^	14.7	104.5	39.3	41.9	15.7	32.3	50.4	11.2%
Mo (mg/kg) ^2^	0.57	4.13	1.48	1.58	0.66	1.2	1.8	40.5%
P (mg/kg) ^1^	230	1340	620	656	251	460	800	12.7%
Pb (mg/kg) ^1^	10.6	1540	52.6	128	205	25.4	146	10.4%
S (%) ^1^	0.01	0.13	0.03	0.03	0.02	0.02	0.04	18.1%
Sb (mg/kg) ^1^	0.6	8.2	1.2	1.5	1.2	0.9	1.7	8.9%
Sc (mg/kg) ^1^	n.d.	24.7	13.3	11.1	6.8	9.4	15.2	4.5%
Sn (mg/kg) ^1^	1.1	64.1	2.2	4.2	7.7	1.7	3.6	12.1%
Sr (mg/kg) ^1^	106	378	232	236	48	206	261	2.3%
W (mg/kg) ^2^	0.7	2.9	1.4	1.4	0.4	1.2	1.6	23.1%
Zn (mg/kg) ^1^	52	6010	120	216	588	90.5	188	8.7%

Notes: 1 = elements analyzed by ICP-AES. 2 = elements analyzed by ICP-MS. n.d. = not detected.

**Table 2:** Summary of elemental concentrations of soil samples collected in Sacramento, CA and analyzed by portable XRF. n.d. = not detected (less than 15 ppm).

Analyte	Min.	Max.	Median	Mean	Standard Deviation
Pb (mg/kg)	n.d.	750	152	171	141
Zn (mg/kg)	67	333	159	167	64

#### 3.1.2. Intercorrelation of Metal Concentrations

The positive correlations of metal concentrations exist where high concentrations of two metals tend to occur together (Table 3). Ninety-one comparisons were made between the available elements. Thirty-two percent of the elements show correlation of 0.5 and higher. Cadmium and copper are correlated with the most other elements. Four of the elements show statistically significant correlation of 0.75 and higher. These pairs include Cd-Pb, Cd-Zn, Pb-Zn, and Sb-Sn.

### 3.2. Spatial Distribution of Metal Concentrations

#### 3.2.1. Semivariance

Log transformed values for Pb concentrations exhibit spatial correlation evidenced by the empirical semivariogram (Figure 1). The semivariogram for lead exhibits a strong degree of spatial correlation, whereas Solt [12] found that the remaining metals, Ag, As, Cu, Mo, Sb, Sn, and Zn, display moderate spatial correlation, reaching a range between 2700 and 8000 m. Bi, Cd, P, Sc, and W do not exhibit spatial correlation at the scale that the ICP samples were collected [12]. It is possible that the spatial correlation does not exist or exists on a smaller scale than the sampled data.

**Table 3:** Correlation matrix of trace metals. Highlighted values represent significantly strong correlation coefficients.

Analyte	Ag	As	Bi	Cd ^*^	Cu	Mo	P	Pb ^*^	S	Sb	Sc	Sn	W	Zn ^*^
Ag	1	0.25	0.47	0.55	0.5	0.21	0.43	0.42	0.26	0.48	0.12	0.47	0.31	0.41
As		1	0.31	0.16	0.48	0.31	0.44	0.28	0.03	0.44	0.21	0.14	0.32	0.19
Bi			1	0.46	0.50	0.34	0.39	0.59	0.22	0.63	0.15	0.69	0.44	0.43
Cd				1	0.56	0.53	0.51	0.79	0.58	0.53	−0.1	0.61	0.38	0.82
Cu					1	0.49	0.51	0.46	0.37	0.63	0.22	0.58	0.52	0.51
Mo						1	0.41	0.44	0.44	0.63	0.16	0.52	0.50	0.39
P							1	0.35	0.37	0.39	0.10	0.33	0.31	0.48
Pb								1	0.41	0.57	−0.2	0.54	0.23	0.75
S									1	0.34	−0.2	0.34	0.35	0.61
Sb										1	0.08	0.75	0.42	0.54
Sc											1	0.08	0.27	−0.1
Sn												1	0.47	0.54
W													1	0.44
Zn														1

^*^ correlation values based on *n* = 102 between elements Cd, Pb, and Zn.

#### 3.2.2. Prediction Map

**Figure 3:**
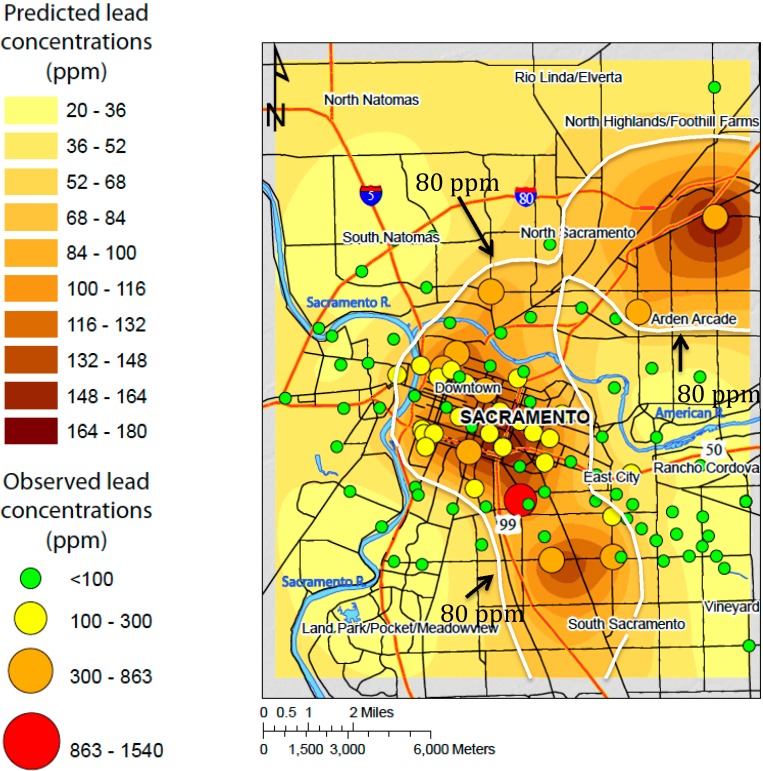
Prediction map of interpolated Pb concentrations in soil (filled contours) kriged using observed Pb concentrations in ssoil (red, yellow, orange, and green circles). Greater concentrations are observed and predicted in the older, downtown area of Sacramento. White line indicates the 80 ppm contour, which the revised CalEPA soil guideline.

The prediction map for Pb exhibits elevated concentrations near the central, downtown areas of Sacramento southeast of the confluence of the American and Sacramento Rivers (Figure 3). The greatest concentrations predicted by ordinary kriging occur in the central older areas of Sacramento. This pattern is consistent with elevated metal concentrations in urban soils that are associated with anthropogenic sources, and similar maps for the other metals reflect the correlations evident in Table 3 [12]. Several studies have demonstrated lead contamination in urban areas is a function of traffic density and age of the urban area [1,2,29,30]. Wang *et al.* [31], used principal component analysis to link elevated Pb and Zn concentrations, which were attributed to vehicle traffic. Diawara *et al.*, [23], used kriging to map predicted concentrations of arsenic, cadmium, lead, and mercury in surface soils from Pueblo (CO, USA). Although Diawara’s analysis revealed varied spatial distribution among the metals, lead contamination was focused around industrial smelting operations. It is important to note that a substantial portion of Sacramento lies within the 80 mg/kg contour (Figure 3), which signifies the newly revised maximum guideline from CalEPA [32].

Distribution of concentrations of Sb, Sn, Sn, Zn, As, and Mo are similar to that of Pb. This includes elevated predicted concentrations spanning the central regions of the study area, which includes downtown Sacramento and areas to the northwest. As in Sacramento, soil contamination in urban areas has been attributed to vehicle traffic and industrial activities cited by several studies [33,34]. Ore mining has also lead to the observation of links among copper, lead, and zinc [19,35].

Variance maps of the spatially correlated elements generally show agreement between predicted and observed concentrations in the central portion of the study area where sample sites are evenly spaced and not isolated (Figure 1) [12]. Variability generally increases where sample sites are isolated and elevated concentrations coincide because isolated samples with high concentrations cause the kriging calculations to weight the isolated points heavily.

## 4. Discussion

Lead is a good indicator of legacy trace metals from anthropogenic sources because of its persistence in the environment. Its relative insolubility at normal pH ranges reduces its mobility in the environment. Whether from gasoline additives, in the case of lead, or engine and tire wear for Cd, Cu, Mo, and Zn, these elements are often associated with anthropogenic input into the environment through automobile traffic [20,32,33,36,37].

Statistical analysis of lead and other trace metals in soils of Sacramento indicates an anthropogenic origin. Box plots showing the statistical distribution of concentrations of each element for all sites reveal non-normal distributions indicating anthropogenic origin. In addition, the presence of elevated concentrations and high standard deviations among the data indicate contamination as opposed to background concentrations of metals. Non-normally distributed metals concentration data and the combination of high concentrations and high standard deviations are often indicators of anthropogenic inputs [24,38]. Statistical comparison between concentration values at each site revealed very high correlation between Cd-Pb, Cd-Zn, Pb-Zn, and Sb-Sn. The co-location of elevated concentrations indicates similar sources for the elemental presence. The presence of lead, cadmium, and zinc have been associated with anthropogenic inputs [20,32,33,36,37].

Ordinary kriging was used to model the spatial distribution of each of the trace metals that exhibit spatial correlation. Ag, Cu, Pb, Sb, Sn, and Zn exhibit common areas of elevated concentrations relative to background levels [12,13]. Results of ordinary kriging of these elements show elevated concentrations in the central, downtown area of Sacramento. These findings imply that the source of these elevated levels of trace metals in soils is, or at one time was, located in the central downtown area of Sacramento. Common sources of lead contamination, such as industrial activity and dense vehicle traffic, are both spatially and temporally concentrated in this part of Sacramento. Lead contamination in urban areas has been the focus of several studies which have linked it to the age of an area and traffic density [1,2,8,30,39,40,41]. The central, downtown area of Sacramento has existed since before the gold rush (1849) as a hub for industry and commerce. Additional samples analyzed by XRF for lead and zinc confirm the initial findings of the MEMS data which show the occurrence of elevated concentrations of these metals in the central, downtown area of Sacramento. The XRF samples were collected in an area identified to have generally elevated concentrations of lead and zinc from the MEMS data. Box plots of soil samples analyzed for lead and zinc by the XRF method show a higher average concentration than lead and zinc from the MEMS method. The XRF data was used only to validate predictions of the existing data set and was not used in the kriging process.

**Figure 4:**
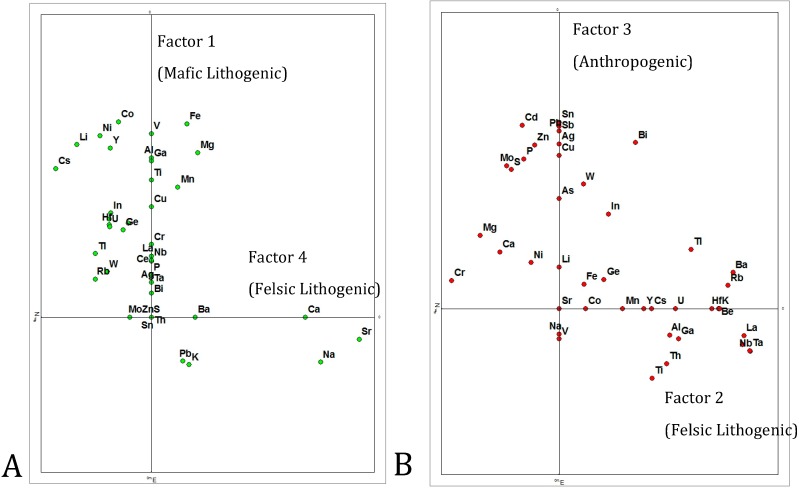
Factor analysis of Sacramento soil geochemical data, suggesting lithogenic and anthropogenic geochemical associations.

Factor analysis of the geochemical data show that the metals are well clustered with four factors accounting for 74% of the variability (Figure 4). Based on major and trace element profiles, Factor 1, with high Al, As, Cu, Fe, Mg, and others, is interpreted as representing mafic lithogenic input, whereas Factors 2 and 4, with high alkalis, are interpreted as representing felsic lithogenic input. These are consistent with earlier findings for the region [20]. Additionally, Factor 3, accounting for 18.4% of the variability, is interpreted as an anthropogenic signal, being dominated by Ag, As, Bi, Cd, Cu, In, Mo, P, Pb, S, Sb, Sn, W, and Zn. In addition to the general association of these elements with anthropogenic input, the overall high values and high standard deviations of these elements also suggests anthropogenic sources [31].

These results show Pb concentrations somewhat lower than soil Pb found associated with houses known to have been painted with Pb paint [39]. In that study, median soil concentrations were about 200 mg/kg in Sacramento. The current study aimed to sample ambient soil conditions, and so samples were not located close to houses that could act as sources of Pb. This reflects the fact that while house-proximal soil Pb may be attributable to Pb paint used on houses, in contrast, ambient soil Pb tends to be the result of legacy Pb emissions, largely from automobiles. Mielke *et al.* [42] estimated that over 10^7^ kg of Pb were emitted in Sacramento — this likely represents a major source of the diffuse soil Pb that remains dispersed throughout the city today. This has clearly elevated ambient soil Pb levels markedly, with a great proportion of the Sacramento area predicted to have soil Pb levels above the CalEPA 80 ppm limit (Figure 3).

The collection, analysis, and subsequent mapping of lead and other trace metals in soils in Sacramento identifies the central, downtown area of Sacramento to have elevated concentrations relative to other areas of the city. This indicates anthropogenic input which could be related to some combination of the temporal persistence and density of traffic and/or industry. The method used in this study for analyzing lead and other trace metal concentrations in urban environments is applicable in areas with similar circumstances such as areas of temporal persistence and high density of traffic and industry as long as a background concentration can be established. This approach can identify areas where concentrations are elevated while testing relationships among metals to identify potential sources. The spatial distribution of trace metals Ag, Cu, Sb, Sn, and Zn are similar to that of lead. Further analysis of this pattern may reveal more about the sources contributing to the concentration of lead and other metals in downtown Sacramento.

This study demonstrates how, like in many cities around the world, the legacy of Pb contamination remains a significant health hazard in Sacramento. Recent work suggests that nearly 10 million children in the U.S. have blood Pb in the range 2–10 µg/dL [43]. Although many children affected by this epidemic may not exhibit symptoms of acute toxicity, even levels below the newly-adopted CDC reference level of 5 µg/dL [44] are associated with neurological and other damage, and even mortality in adults [44,45,46]. Soil Pb is now well known to be a major source of exposure, particularly for children, even well below current EPA action levels of 400 mg/kg [10,47,48]. Although EPA is engaged in superfund cleanups of many Pb-related sites around the country, zero CDC funding is currently assigned to assess the health impacts of those sites and the cleanups. The plethora of evidence on Pb hazards, particularly in urban environments, calls for a national strategy on addressing this quiet epidemic among urban children in the U.S.

## 5. Conclusions

Non-normal abundance distributions and geospatial correlations indicate anthropogenic origins of several heavy metals in Sacramento soils. Cd, Pb, and Zn are highly correlated, as are Sb and Sn. In addition, Ag, As, Cu, and Mo show spatially correlated distributions. Heavy metals are in general elevated in older, central Sacramento where vehicle traffic and industry have historically been dense.

Lead in soil, with its ability to negatively impact human health at low levels, remains a major concern. The utility of predicting the spatial distribution of lead concentrations is evident in the residential and abatement decision-making processes. In addition, the comparison of the spatial distribution of other trace metals (Ag, Cu, Sb, Sn, and Zn) from soils can indicate relationships among lead and these trace metals. This knowledge can aide in determining possible sources of lead contamination, which, in turn, will better allow for the protection of human health.
